# Development of a reverse genetics system for West Nile virus (Kunjin type)

**DOI:** 10.3389/fvets.2025.1671591

**Published:** 2025-08-25

**Authors:** Zhen Wu, Tao Hu, Zhou Zhou, Yu He, Tao Wang, Mingshu Wang, Renyong Jia, Dekang Zhu, Mafeng Liu, Xinxin Zhao, Qiao Yang, Ying Wu, Shaqiu Zhang, Juan Huang, Xumin Ou, Di Sun, Bin Tian, Anchun Cheng, Shun Chen

**Affiliations:** ^1^Institute of Veterinary Medicine and Immunology, Sichuan Agricultural University, Chengdu, Sichuan, China; ^2^Research Center of Avian Disease, College of Veterinary Medicine, Sichuan Agricultural University, Chengdu, Sichuan, China; ^3^Key Laboratory of Animal Disease and Human Health of Sichuan Province, Sichuan Agricultural University, Chengdu, Sichuan, China; ^4^Key Laboratory of Agricultural Bioinformatics of Ministry of Education, Sichuan Agricultural University, Chengdu, Sichuan, China

**Keywords:** Kunjin virus, reporter replicon, packaging system, reporter virus, reverse genetics system

## Abstract

Kunjin virus (KUNV), a naturally attenuated strain of West Nile virus (WNV), shares similar transmission modes and hosts—primarily mosquitoes, birds, and horses. Globally, reverse genetics is the principal methodology for characterizing the molecular etiology of flaviviruses. In this study, cytomegalovirus (CMV) promoter-driven KUNV reporter replicons were engineered to incorporate three distinct reporter genes: Nanoluc, oxGFP, and mCherry. These replicons demonstrated successful translation and replication in mammalian (BHK-21), avian (DEF), and avian hepatic (LMH) cell lines. Additionally, an *in vitro* pseudovirus packaging system for KUNV was established using helper plasmids expressing either full-length C-prM/E or a truncated C-terminal variant (C_18_-prM/E). Both plasmids efficiently packaged replicon RNA into pseudoviruses, with C_18_-prM/E showing significantly higher packaging efficiency than full-length C-prM/E. Furthermore, leveraging a previously developed full-length infectious KUNV clone, a stable reporter virus was generated by inserting the NanoLuc luciferase gene. The reporter virus maintained genomic integrity over five serial passages with no loss of the reporter gene. Collectively, these experiments establish robust *in vitro* reverse genetics systems for KUNV. These tools constitute valuable molecular resources for investigating the KUNV lifecycle, advancing antiviral drug screening, and facilitating vaccine development.

## Introduction

Kunjin virus (KUNV), a naturally attenuated strain of West Nile Virus (WNV) (1, 2), was first isolated from *Culex annulirostris* in Australia in the year 1960 (3), and it belongs to the family Flaviviridae. The Flavivirus genus consists of more than 70 viruses, such as yellow fever virus (YFV), dengue virus (DENV), Japanese encephalitis virus, and zika virus (ZIKV). Like other members of the genus Flavivirus, KUNV have a positive-strand RNA genome of 11,022 nucleotides and genome contains a single open reading frame (ORF) that encodes a polyprotein, which is translated and modified to cleave into three structural proteins—capsid protein (C), membrane protein (M), and envelope protein (E)—and seven non-structural proteins: NS1, NS2A, NS2B, NS3, NS4A, NS4B, and NS5 (4). The ORF is flanked by 5’UTR and 3’UTR, consisting of 96 and 624 nucleotides, respectively (5). Structural proteins are involved in the invasion, assembly and release of virions within host cells (6, 7). Nonstructural proteins are involved in viral genome replication and virion assembly. The untranslated region (UTR) is involved in viral replication and host adaptation (8, 9).

KUNV is the causative agent of a human disease characterized by a febrile illness with rash or mild encephalitis and occasionally by a neurological disease in horses (10). WNV first appeared in Africa, and there are two main lineages: lineage I and lineage II, among which lineage I contains KUNV isolates (11). KUNV is widely distributed in Australia (12), and humans could be infected by mosquito bites. The clinical symptoms of KUNV are mainly mild fever, headache, myalgia, and rash; however, compared with WNV, KUNV is rarely isolated from humans and rarely associated with severe diseases in humans (13–15). KUNV and WNV share a natural transmission cycle between Culex mosquito vectors and avian hosts. Some birds and mammalian hosts can be used as the vertebrate hosts of KUNV (16, 17). KUNV hosts also include poultry, pigs, cattle, and horses, and humans and horses are considered to be the terminal hosts (18).

In this study, we delineate CMV promoter-driven KUNV reporter replicons, each carrying three distinct reporter genes (Nanoluc [Nluc], oxGFP, or mCherry). These replicons exhibited normal replication and translation profiles in BHK-21, Duck embryonic fibroblasts (DEF), and Leghorn male hepatoma (LMH) cells. Furthermore, we engineered a packaging system for KUNV, yielding single-round infectious particles (SRIPs) housing viral subgenomic replicon DNA. Moreover, we successfully engineered a reporter virus expressing the Nluc gene, demonstrating stable proliferation in BHK-21 and DEF cells. To summarize, our work culminates in the development of a comprehensive suite of reverse-genetics molecular tools, including KUNV reporter replicons, a packaging system, and a reporter virus. These tools serve as a foundational platform for further investigation into KUNV replication dynamics and the viral life cycle.

## Materials and methods

### Cells

Baby hamster kidney (BHK-21; ATCC, CCL-10) cells were cultured in Dulbecco’s modified Eagle’s medium (DMEM; Gibco, Shanghai, China) supplemented with 10% fetal bovine serum (FBS; Gibco, New York, USA) and incubated at 37°C with 5% CO_2_. DEF were prepared from 10-day-old duck embryos, propagated into DMEM supplemented with 10% newborn calf serum (NBCS; Gibco), and incubated at 37°C with 5% CO_2_. LMH (ATCC, CRL-2117) cells were cultured in DMEM/F12 supplemented with 10% FBS and incubated at 37°C with 5% CO_2_.

### Reporter replicon plasmid and package helper plasmid construction

**Figure 1:**
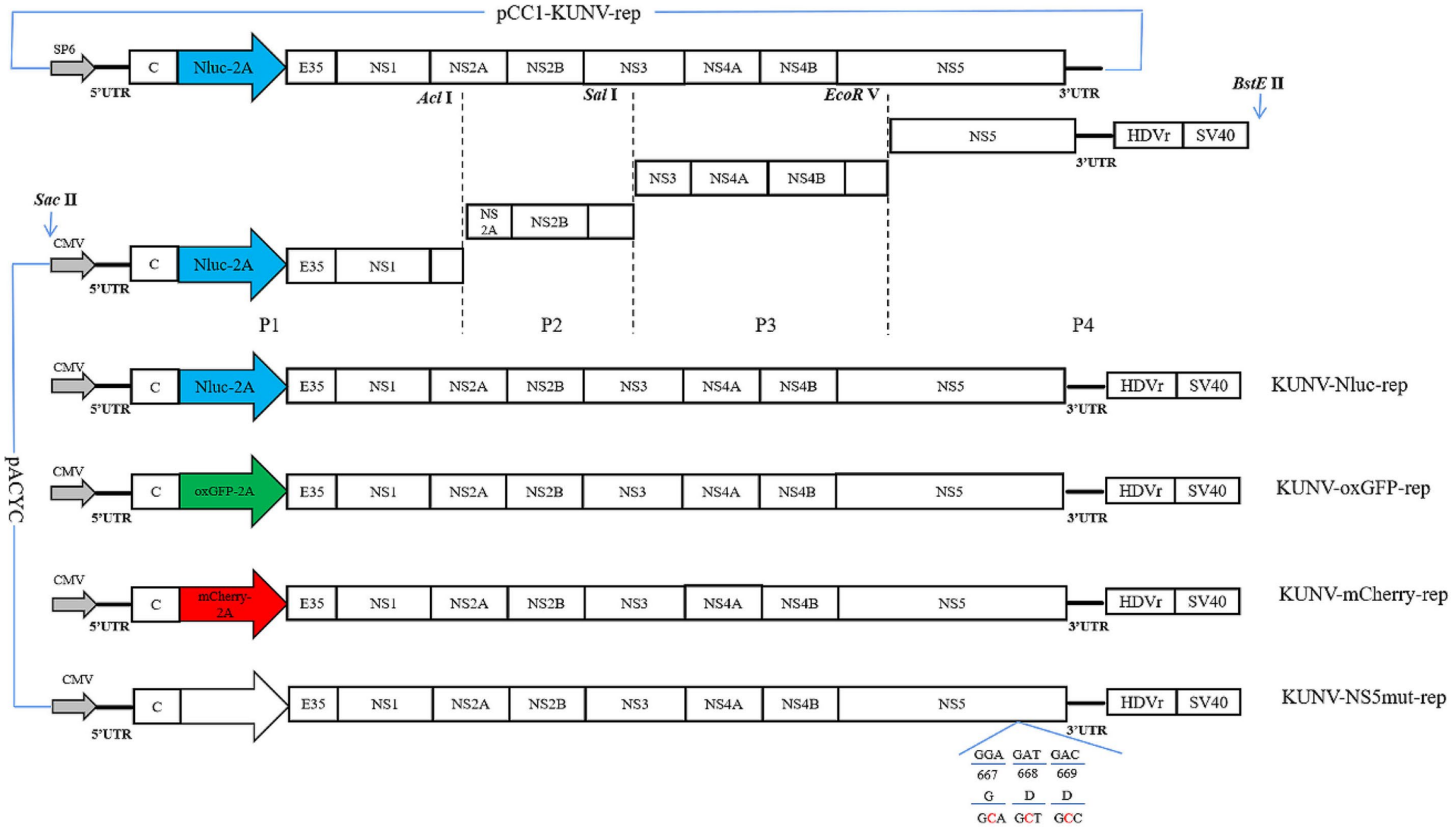
Construction of the replicon of KUNV. Schematic diagram for the design of the genomes of KUNV reporter replicon. The schematic diagram for the construction of replicons: KUNV-Nluc-rep, KUNV-oxGFP-rep, KUNV-mCherry-rep, and KUNV-NS5mut-rep. UTR: untranslated regions; 2A: foot-and-Mouth Disease Virus 2A Peptide; HDVr: hepatitis delta virus ribozyme; SV40: simian virus 40 polyadenylation signal. The last 35 amino acids of the E protein and intact C protein are retained.

Overlap extension PCR technology and homologous recombination strategy were used to construct the KUNV reporter replicons. Two restriction enzyme sites SacII and BstEII were selected in the pACYC177 vector, and three restriction enzyme sites EcoRV, SalI, and AclI were selected in the KUNV replicon genome for subsequent restriction ligation. Plasmid pACNR CQW1-intron was used to clone the cytomegalovirus (CMV) promoter (directly transfected into cells without *in vitro* transcription) and hepatitis delta virus ribozyme (HDVr) and Simian virus 40 (SV40) polyadenylation tail signal sequence, which was engineered for transcription termination. The CMV and the “5’UTR-EcoRV” fragment were fused into P1 fragment, and the P1 fragment covered the gene sequence between the “SaclI-CMV-5’ UTR-EcoRV.” Fragment P2 covered the gene sequence between “EcoRV- SalI.” Fragment P3 covered the gene sequence between “SalI- AclI.” HDVr and SV40 poly (A) were fused with “SalI-3’UTR” to form a complete P4 fragment covering the gene sequence between “SalI-BstElI.” Then, the P4 fragment was homologously recombined with the linearized pACYC177 vector to form a subcloning plasmid pACYC-P4. Then, the pCC1-KUNV-rep (Courtesy of Professor Andres Merits, University of Tartu, Estonia) was used as the template to amplify the KUNV genome sequence, and the P1-P4 was ligated into the pACYC177 vector using EcoRV, SalI, and AclI restriction enzymes. Finally, KUNV replicon plasmids that carried three different reporter genes (Nluc, oxGFP, and mCherry) were constructed (Figure 1) and named KUNV-Nluc-rep, KUNV-mCherry-rep, and KUNV-oxGFP-rep. Defected replicons with inactivated NS5-GDD motifs were generated simultaneously and named KUNV-Nluc-NS5mut-rep, KUNV-mCherry-NS5mut-rep, and KUNV-oxGFP-NS5mut-rep.

The packaging helper plasmid pcDNA3.1-CprME was synthesized by Shanghai Sangon Biological Engineering Co., LTD Shanghai, China. Plasmid pcDNA3.1-CprME was used to clone pcDNA3.1-C_18_prME (retaining the 18 amino acids of the Capsid terminal). The C_18_prME gene was amplified and ligated into the pCDNA3.1(+) linear vector after restriction enzyme digestion with HindIII and AplI.

### Construction of reporter virus

**Figure 2:**
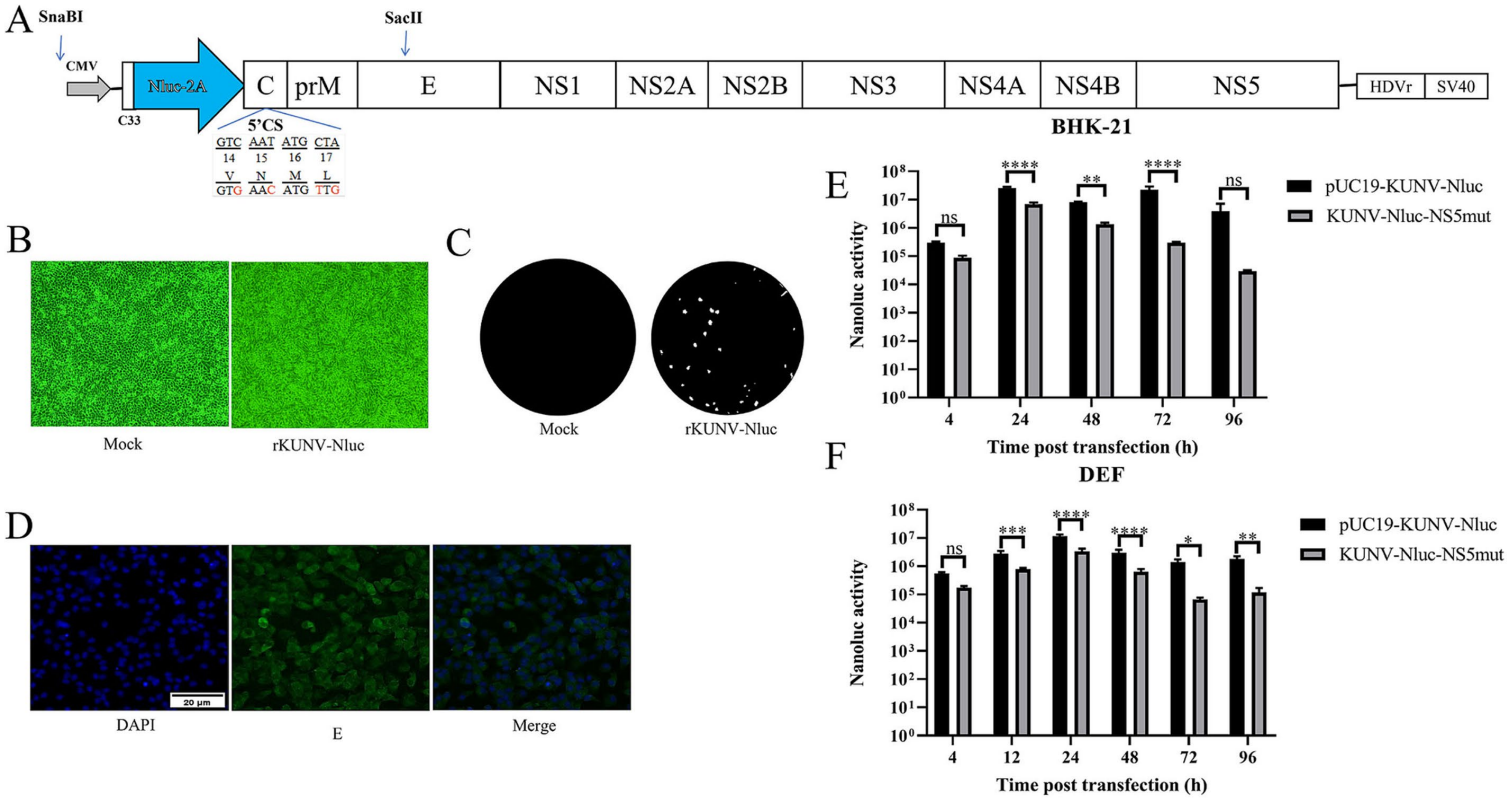
Construction of reporter KUNV expressing Nluc luciferase. **(A)** The schematic diagram for the construction of rKUNV-Nluc. C33: the first 33 aa of the C protein; 2A: foot-and-Mouth Disease Virus 2A Peptide; HDVr: hepatitis delta virus ribozyme; SV40: simian virus 40 polyadenylation signal. **(B)** CPE were observed following transfection of BHK-21 cells with the KUNV infectious clone. **(C)** Plaque morphology of rKUNV-Nluc on BHK-21 cells. **(D)** Post-transfection with rKUNV-Nluc DNA, positive fluorescence was detected by using mouse anti KUNV E protein polyclonal antiserum as the primary antibody. **(E,F)** Luciferase activity kinetics of BHK-21 or DEF cells transfected with rKUNV-Nluc DNA. *p < 0.05, **p < 0.01, ***p < 0.001, ****p < 0.0001, and ns represents no significance.

According to the construction strategy of DTMUV reporter virus (19), the design scheme of reporter virus rKUNV-Nluc is shown in Figure 2A. Based on the full-length infectious clone of KUNV constructed in our laboratory (20), the Nluc luciferase reporter gene was inserted into the genome of KUNV using SnaBI and SaclI double restriction sites. The Nluc gene was inserted between the 5’UTR and the capsid protein, and the first 33 amino acids (C33) of the Capsid protein of the Nluc gene upstream were retained. The C33 gene sequence contained the necessary elements for genome cyclization. At the same time, the 5’CS (amino acids 14–17 of Capsid protein) of the complete capsid gene of Nluc gene downstream was silent mutations to limit the long-range interaction with 3’CS.

### Transfection

BHK-21, DEF, or LMH cells were seeded in 24-well plates for transfection. After 16 h of cell culture (80–90% confluence), cells were transfected with 0.5 μg DNA per well using Hieff Trans™ Liposomal Transfection Reagent (YEASEN Biotech Co., Ltd), according to the manufacturer’s instructions. After transfection, the cells were incubated at 37°C with 5% CO_2_. For rKUNV-Nluc, the supernatant was not harvested until an obvious cytopathic effect (CPE) appeared, and then, it was used for the next infection.

### Virus titration, growth curve and plaque assay

As previously described He et al. (21), viral titers were determined by the median tissue culture infectious dose (TCID_50_) method on BHK-21 cells. The viral sample was serially diluted 10-fold in DMEM, and then, 100 μL dilutions of the viral sample were distributed to each of the 8 wells of a 96-well plate seeded with monolayer BHK-21 cells. After 120 h incubation at 37°C with 5% CO_2_, cytopathic effect was examined by using microscopy, and viral titers were calculated using the Karber method.

BHK-21 cells and DEF cells were seeded in 24-well plates, and the cells were infected with rKUNV-Nluc at 100 TCID_50_. Every 24 h, the supernatant was collected and subjected to viral titration as described above. Simultaneously, after the supernatant was harvested, the cell monolayer was washed once with phosphate-buffered saline (PBS) and then lysed using the Glo lysis buffer (Promega, WI, USA) at room temperature for 5 min. The cells were scraped from the plates and stored at −80°C for the next luciferase activity assay.

For the plaque assay, viral samples were 10-fold serially diluted in DMEM; 300 μL samples of each dilution were added to a 6-well plate seeded with BHK-21 cells (80–90% confluence). Then, the samples were incubated for 1.5 h and swirled every 15 min to ensure viral attachment. After the incubation, 2 mL of 0.75% methyl cellulose overlay containing 2% FBS and 1% penicillin/streptomycin was added to each well, and the plate was incubated at 37°C for 6 days. Then, methyl cellulose overlay was removed, the plate was washed thrice with PBS, and fixed with 4% formaldehyde at room temperature for 20 min. After removing the fixative, the plate was stained with 1% crystal violet for 40 s, the cells were washed carefully, and visible plaques were observed.

### Nluc luciferase activity assay

For the detection of Nluc activity, cell samples were harvested at the times indicated in the figures or figure captions. Cells were lysed with 100 μL of Glo lysis buffer; the resulting lysates were vortexed thoroughly, and 20 μL aliquots were taken for luciferase activity assays. To detect Nluc activity, a Nano-Glo Luciferase Assay System (Promega) and a GloMax Navigator System (Promega) were used according to the manufacturer’s instructions. Then, 50 μL (mixing Nano-Glo substrate and Nano-Glo assay buffer at a ratio of 1:49) of Nano-Glo Luciferase Assay Reagent was added to 20 μL of the sample in a light-tight 96-well tissue culture plate. The solution was mixed for optimal consistency and then detected by the microplate luminescence detector. The absolute values of luciferase activity were used for data analysis.

### Fluorescent protein expression assay

Cell slides were collected from 24-well plates, which transfected with KUNV-mCherry-rep and KUNV-oxGFP-rep, at different time points, as well as from plates infected with the KUNV SRIPs supernatant. Cell slides were fixed with 4% paraformaldehyde for 1 h at 4°C, and then permeabilized for 1 h at 4°C with 0.25% Triton in PBS. Each of the above steps was followed by two washes with PBS. Finally, cells were stained with DAPI in PBS for 15 min, and fluorescence images were acquired using a fluorescence microscope (Nikon, Tokyo, Japan).

### Packaging system assay

To construct an efficient packaging system for KUNV, two DNA-based helper replicon plasmids, expressing full-length capsid protein and truncated capsid protein, were designed. Plasmids pCDNA3.1-CprME and pCDNA3.1-C_18_prME, encoding the polyproteins C-prM-E and C_18_-prM-E, were transfected into cells to supply prM and E proteins *in trans,* respectively, forming viral particles. When BHK-21 cells in a 6-well plate reached 80–90% confluence, equal amounts of KUNV reporter replicon and pCDNA3.1-CprME or pCDNA3.1-C_18_prME plasmids (helper packaging plasmid and replicon in a ratio of 1:1) were co-transfected into BHK-21 cells using Hieff Trans™ Liposomal Transfection Reagent (YEASEN Biotech Co., Ltd), according to the manufacturer’s instructions. After transfection, the cells were incubated at 37°C with 5% CO_2_ for 48 h. The KUNV SRIPs in the supernatant were harvested and stored at −80°C.

To verify the packaging efficiency, BHK-21 cells were seeded in 24-well plates, and when the cells reached approximately 80–90% confluence, the cell culture medium was removed and washed three times with PBS. The cells were then infected with KUNV-SRIPs harvested in the previous step and incubated at 37°C with 5% CO_2_ for 1.5 h. Afterward, the supernatant was removed and replaced with cell culture maintenance medium (DMEM with 2% FBS). Forty-eight h post-infection, the expression of luciferase and fluorescent protein was detected.

### Indirect immunofluorescence assay

Cells were washed twice with PBS, fixed with 4% paraformaldehyde for 1 h at 4°C, and then permeabilized with 0.25% Triton in PBS for 1 h at 4°C. After a 1-h incubation at 37°C in a blocking buffer containing 5% bovine serum albumin (BSA) in PBS, cells were treated with mouse dsRNA J2 antibodies for 2 h and then incubated with either FITC fluorescently labeled goat anti-mouse IgG or TRITC fluorescently labeled goat anti-mouse IgG for 1 h. Finally, cells were stained with DAPI in PBS for 15 min. Each step was followed by washing the cell thrice with ice-cold PBST (1‰ Tween-20 in PBS) for 5 min in an orbital shaker. Fluorescence images were acquired under a fluorescence microscope. For rKUNV-Nluc, cells were treated with mouse anti KUNV E protein polyclonal antiserum as primary antibodies for 2 h.

### Virulence in duck embryos

All duck embryos were purchased from the Waterfowl Breeding Center of Sichuan Agriculture University and randomly divided into three groups. 10-days-old embryos eggs per group were injected with 100 μL or 500 μL rKUNV-Nluc dilution and 100 μL DMEM by allantoic cavity inoculation at 10^3.5^ TCID_50_. DMEM was used to dilute the virus stocks to the desired concentration. The eggs were incubated continuously at 37°C and checked daily using an egg candler. If the embryos lose movement and blood vessels were desquamated, the embryos eggs were regarded as dead. The survival time of the inoculated eggs was noted.

### Data statistical analysis

Statistical analyses were performed with GraphPad Prism 5 (GraphPad Software Inc., San Diego, CA, USA). The differences between the values were evaluated by Student’s *t*-test. A *p* < 0.05 was considered statistically significant, and all data are expressed as the mean ± SEM.

## Results

### Construction of KUNV reporter replicons

Figure 1 depicts KUNV reporter replicons engineered to encode Nluc luciferase or the fluorescent proteins oxGFP and mCherry. Subgenomic fragments (P1-P4) were cloned using the pCC1-KUNV-rep plasmid as the template. Individual replicons containing the Nluc, mCherry, and oxGFP reporter genes were generated by overlapping PCR (Table 1). Additionally, a replication-defective control replicon, containing an inactivated NS5 GDD catalytic motif, was constructed to confirm that reporter gene expression was dependent on functional viral replication.

**Table 1:** Primers for construction of reporter replicon plasmids.

Name	Primers (5′-3′)
KUNV-P1 F	AACGGCTTTGCCGCGGGTGATGCGGTTTTGG
KUNV-P1 R	AGATGTTGTGGTGAACGTTATCGCTCTCAA
KUNV-P2 F	ACGGCTTTGCCGCGGAACGTTCACCACAAC
KUNV-P2 R	GGGTTGCATGGCACATAACGTCGACGATCTCAT
KUNV-P3 F	ACGGCTTTGCCGCGGCGTCGACGTTATGTG
KUNV-P3 R	TGAGGACTCTCCGATATCACAAAGGAGAGT
KUNV-P4 F	GACACTCTCCTTTGTGATATCGGAGAGTCC
KUNV-P4 R	ACAGCCGACAGGATGGTGACCGATTAAGATACATTG

### Functional analysis of KUNV reporter replicons in mammalian and avian cells

**Figure 3:**
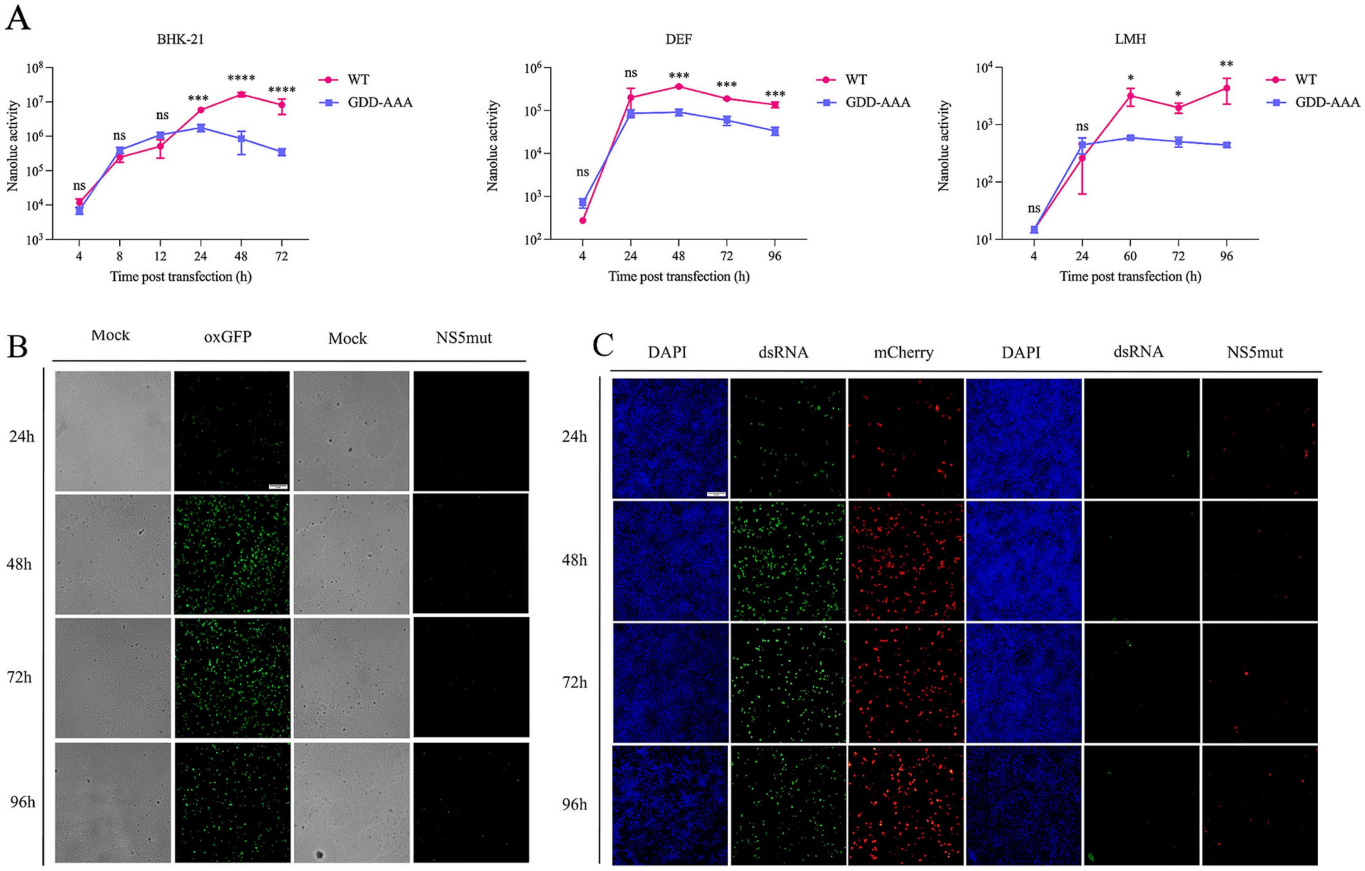
Validation the replication of KUNV replicon in different cells. **(A)** Replicon assay for KUNV-Nluc-rep in BHK-21, DEF, and LMH cells. For BHK-21 cells transfected with the replicon, cells were lysed and subjected to luciferase activity assay at 4, 8, 12, 24, 48, and 72 hpt. For DEF cells transfected with the replicon, cells were lysed and luciferase activity was measured at 4, 24, 48, 72, and 96 hpt. For LMH cells transfected with the replicon, cells were lysed and luciferase activity was assayed at 4, 24, 60, 72, and 96 hpt. The background of the assay was determined with the active site NS5 polymerase mutant (GDD-AAA); **(B,C)** oxGFP and mCherry expression from KUNV replicon vectors. BHK-21 cells were transfected with plasmids KUNV-mCherry-rep, KUNV-NS5mut-rep for 24 h, 48 h, 72 h, and 96 h. Meanwhile, the replication of KUNV-mCherry-rep was monitored by detecting the production of dsRNA. *p < 0.05, **p < 0.01, ***p < 0.001, ****p < 0.0001 and ns represents no significance, Scale bar = 20 μm.

The reporter replicon plasmids (KUNV-Nluc-rep, KUNV-mCherry-rep, and KUNV-oxGFP-rep) were transfected into mammalian BHK-21 cells, avian DEF cells, and avian hepatic LMH cells. Reporter gene expression was monitored through luciferase activity or fluorescence intensity (Figure 3). In the KUNV-Nluc-rep group, luciferase activity increased significantly from 4 to 48 h post-transfection (hpt), peaking at 48 hpt before declining at 72 hpt. Activity consistently exceeded that of the catalytically defective NS5mut control at 24 hpt (*p* < 0.001) and 48 hpt (*p* < 0.0001) (Figure 3A, left). oxGFP fluorescence exhibited similar kinetics, with maximal signal intensity at 48 hpt (Figure 3B). No specific fluorescence was detected in NS5mut. dsRNA immunofluorescence confirmed replication competence: KUNV-mCherry-rep-transfected cells displayed time-dependent dsRNA accumulation, while NS5mut showed no signal due to impaired RNA-dependent RNA polymerase (RdRp) activity (Figure 3C). In DEF cells, Nluc activity was comparatively weak but demonstrated significant replicon-dependent expression at 48 hpt versus NS5mut (*p* < 0.001) (Figure 3A, middle). In LMH cells, despite low baseline activity, KUNV-Nluc-rep showed statistically significant replication relative to NS5mut at 60 hpt (*p* < 0.05) and 96 hpt (*p* < 0.01) (Figure 3A, right). These data demonstrate successful construction of functional KUNV reporter replicons, with validated translation and replication capabilities across mammalian (BHK-21) and avian (DEF, LMH) cell lines.

### Packaging system

**Figure 4:**
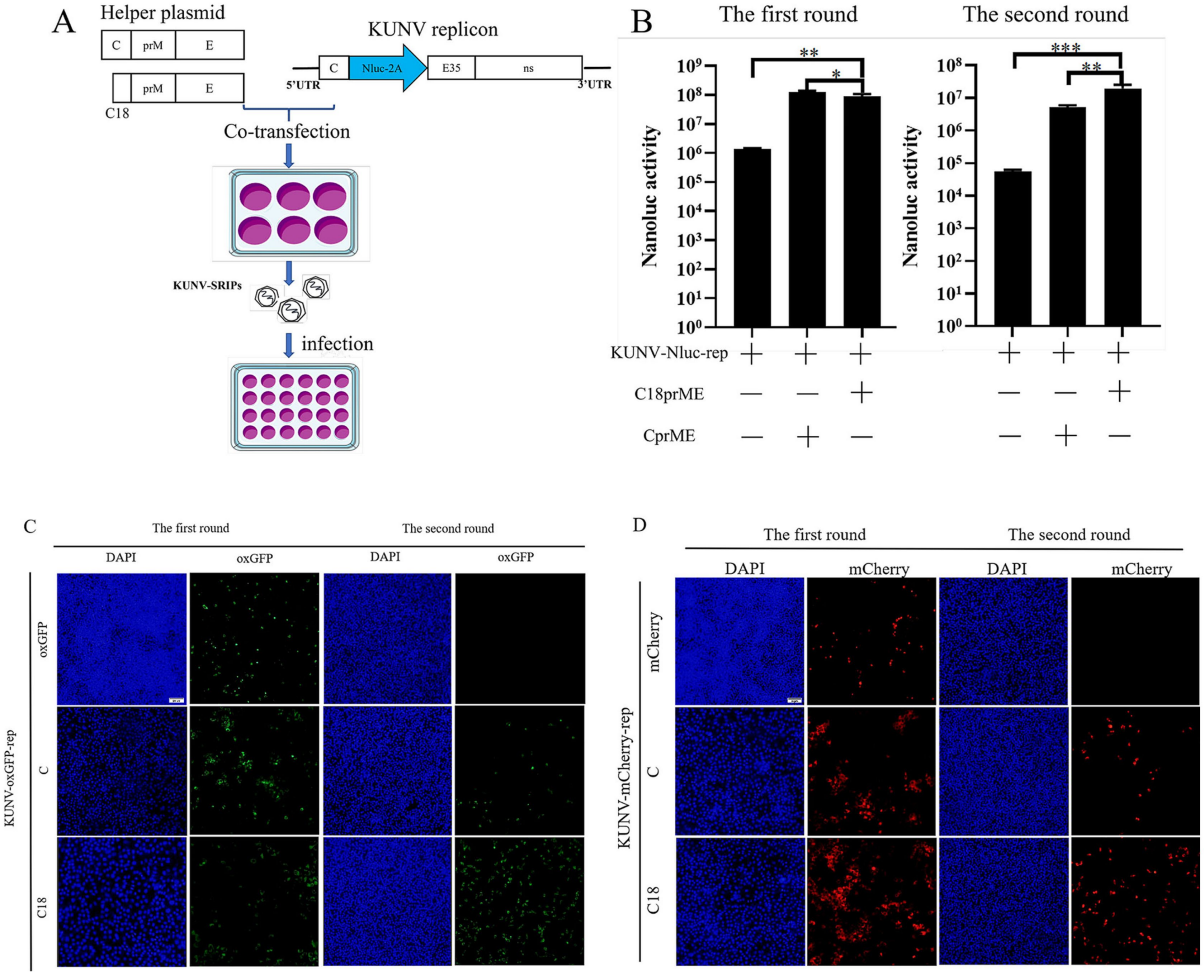
Establishment of the KUNV packaging system. **(A)** Schematic presentation of the experimental procedure used for the production of SRIPs. Following 48 h of co-transfection with helper plasmids (C_18_-prM/E or C-prM/E) and the KUNV-Nluc-rep replicon in the first round, the culture supernatant was collected and used to infect BHK-21 cells. After 48 h of infection, the cell pellets were harvested. The cell pellets from both the first and second rounds were then lysed and analyzed for Nluc luciferase activity. Cells transfected with the replicon alone served as the control. **(C,D)** Following 48 h of co-transfection with helper plasmids (C_18_-prM/E or C-prM/E) and the KUNV-oxGFP-rep **(C)** or KUNV-mCherry-rep **(D)** replicon in the first round, the culture supernatant was collected and used to infect BHK-21 cells. After 48 h of infection, cell samples were collected. Samples from the first round of transfection and the second round of infection were fixed and examined for expression of oxGFP and mCherry proteins by fluorescence microscopy. Cells transfected with the replicon alone served as a control. *p < 0.05, **p < 0.01, ***p < 0.001, Scale bar = 20 μm.

To establish a KUNV single-round infectious particles (SRIPs) packaging system for viral assembly studies, we co-transfected BHK-21 cells with KUNV reporter replicons and structural gene helper plasmids (C-prM/E or C₁₈-prM/E) (Table 2). The supernatants containing putative SRIPs were harvested and used to infect fresh BHK-21 monolayers (Figure 4A). During the initial transfection phase (Figures 4B–D), comparable Nluc luciferase activity and fluorescent protein expression were observed in both the replicon-only control and cells co-transfected with helper plasmids, confirming normal replicon function. Crucially, in the subsequent infection phase (Figures 4B–D), Nluc activity was significantly reduced in cells infected with supernatant from the replicon-only control compared to supernatants derived from co-transfection groups. Furthermore, SRIPs generated using the truncated C₁₈-prM/E helper plasmid demonstrated substantially higher luciferase activity than those incorporating full-length C-prM/E. This enhanced packaging efficiency was corroborated by oxGFP and mCherry fluorescence signals, which were significantly more intense in SRIPs produced with the C₁₈-prM/E construct versus full-length C-prM/E. These results collectively validate successful KUNV SRIPs packaging and demonstrate that the pcDNA3.1-C_18_prME helper plasmid exhibits superior packaging efficiency to pcDNA3.1-CprME.

**Table 2:** Primers for construction of pcDNA3.1-C18prME plasmids.

Name	Primers (5′-3′)
C18prME F	GTTTAAACTTAAGCTTGCCACCATGGGAGGAAAGACCGGA
C18prME R	AGCGGGTTTAAACGGGCCCTTAAGCATGCACGTTC

### Recovery and identification of rKUNV-Nluc

Leveraging a previously established full-length KUNV infectious clone, a reporter virus encoding the Nluc gene was engineered (Table 3). The Nluc gene cassette was inserted into the KUNV genome utilizing SnaBI and SaclI restriction sites, generating the full-length cDNA infectious clone designated pUC19-KUNV-Nluc (Figure 2A). Concurrently, a replication-deficient mutant control clone, pUC19-KUNV-Nluc-NS5mut, was constructed by introducing a catalytically deficient mutation into the NS5 GDD motif. The fidelity of the constructs was confirmed by Sanger sequencing, ensuring the absence of spurious mutations.

**Table 3:** Primers for construction of pUC19-KUNV-Nluc plasmids.

Name	Primers (5′-3′)
pcDNA3.1-reCapsid F	TTTAAACTTAAGCTTATGTCTAAGAAACCA
pcDNA3.1-reCapsid R	CGGGTTTAAACGGGCCCGGGTGATACTAAATC
KUNV-5’CSF	GGCAAAAGCCGGGCTGTGAACATGTTGAAACGCGGAA
KUNV-5’CS R	CAACATGTTCACAGCCCGGCTTTTGCCGGGCCCTCCT
KUNV-C33 Nluc F	GGACTGAAGAGGGCAATGGTCTTCACACTC
KUNV-C33 Nluc R	TGAAGACCATTGCCCTCTTCAGTCC
KUNV-reC33 F	CCCTGGGCCCATGTCTAAGAAACCAGGAGGGCCCG
KUNV-2A-C R	CCTGGTTTCTTAGACATGGGCCCAGGGTTG
KUNV-reCMV F	CTACTTGGCAGTACATCTACGTATTAGTCATC
KUNV-5′UTR-C R	TGGTTTCTTAGACATCGAGATCTTCGTGCT

To rescue recombinant rKUNV-Nluc, BHK-21 or DEF cells at 80–90% confluency were transfected with pUC19-KUNV-Nluc or the replication-deficient pUC19-KUNV-Nluc-NS5mut plasmid. Luminescence signals were detected for both constructs at 4-h post-transfection. In BHK-21 cells, rKUNV-Nluc luminescence exhibited biphasic kinetics: initial time-dependent escalation culminating in a 72-h peak, followed by reduction (Figure 2E). Conversely, NS5mut luminescence progressively declined from 24 h. DEF cells displayed distinct kinetics, with rKUNV-Nluc luminescence peaking at 24 h before diminishing (Figure 2F), while NS5mut demonstrated comparable decay to BHK-21 cultures. Envelope protein expression was independently confirmed by immunofluorescence assay (Figure 2D). Transfected BHK-21 monolayers developed marked cytopathic effect (CPE) by Day 5 (Figure 2B), with plaque assays confirming rKUNV-Nluc produced distinct plaques (Figure 2C). These cumulative findings validate the successful rescue of replication-competent rKUNV-Nluc.

### Characteristics of rKUNV-Nluc

**Figure 5:**
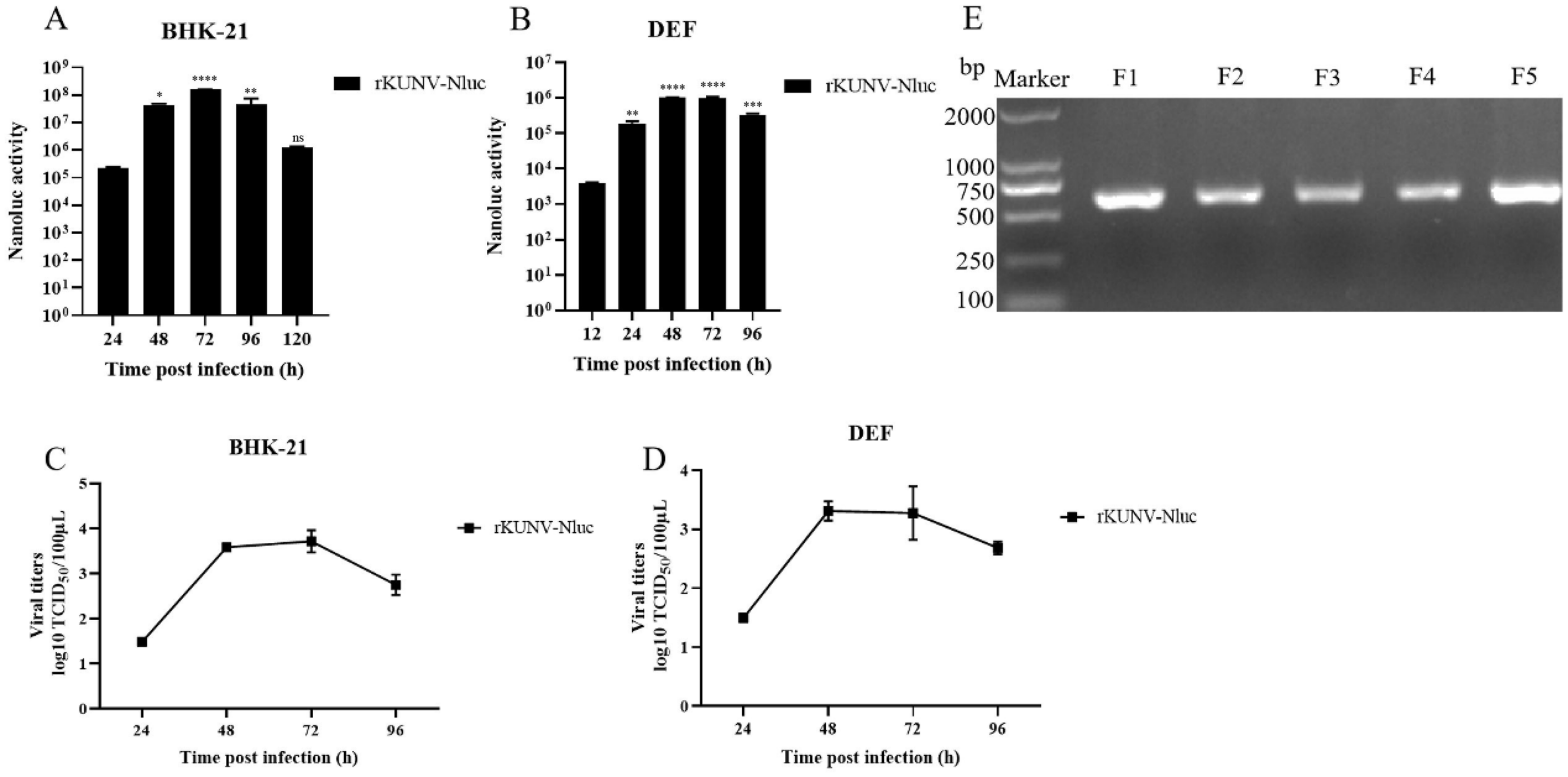
*In vitro* characteristics of rKUNV-Nluc. **(A,B)** Nluc activity was determined following rKUNV-Nluc infection of BHK-21 and DEF cells to represent the growth kinetics of the recombinant virus; **(C,D)** Growth curve of rKUNV-Nluc in BHK-21 or DEF cells; **(E)** Genetic stability of reporter gene of rKUNV-Nluc. The F1-F5 rKUNV-Nluc were suffered RT-PCR, a pair of primers were used to amplifying a fragment (608 bp, in length) covering Nluc luciferase gene. *p < 0.05, **p < 0.01, ***p < 0.001, ****p < 0.0001, and ns represents no significance.

To characterize rKUNV-Nluc *in vitro*, viral growth kinetics and luminescence profiles were assessed in BHK-21 and DEF cells infected with first-passage virus (F1). In both cell lines, Nluc luminescence peaked at 72 hpi before declining (Figures 5A,B). The multi-step growth curve revealed significantly higher titers in BHK-21 cells than DEF cells at all matched time points. Peak titers in BHK-21 cells occurred at 72 hpi, whereas DEF cells exhibited maximum virus production at 48 hpi (Figures 5C,D). These data demonstrate superior replication kinetics of rKUNV-Nluc in mammalian BHK-21 cells compared to avian DEF cells.

To assess reporter gene stability in rKUNV-Nluc, the recombinant virus underwent five serial passages in BHK-21 cells. The RT-PCR analysis of passages F1-F5 confirmed retention of the Nluc gene without deletion (Figure 5E), demonstrating stable maintenance of the reporter in the viral genome across propagation rounds. Genomic sequencing of F5 revealed a single adaptive mutation (NS3-R250L) that initially emerged in F3 and stably propagated through subsequent passages. These findings collectively demonstrate the genomic stability of rKUNV-Nluc during *in vitro* cultivation.

### Virulence of rKUNV-Nluc in duck embryos

**Figure 6:**
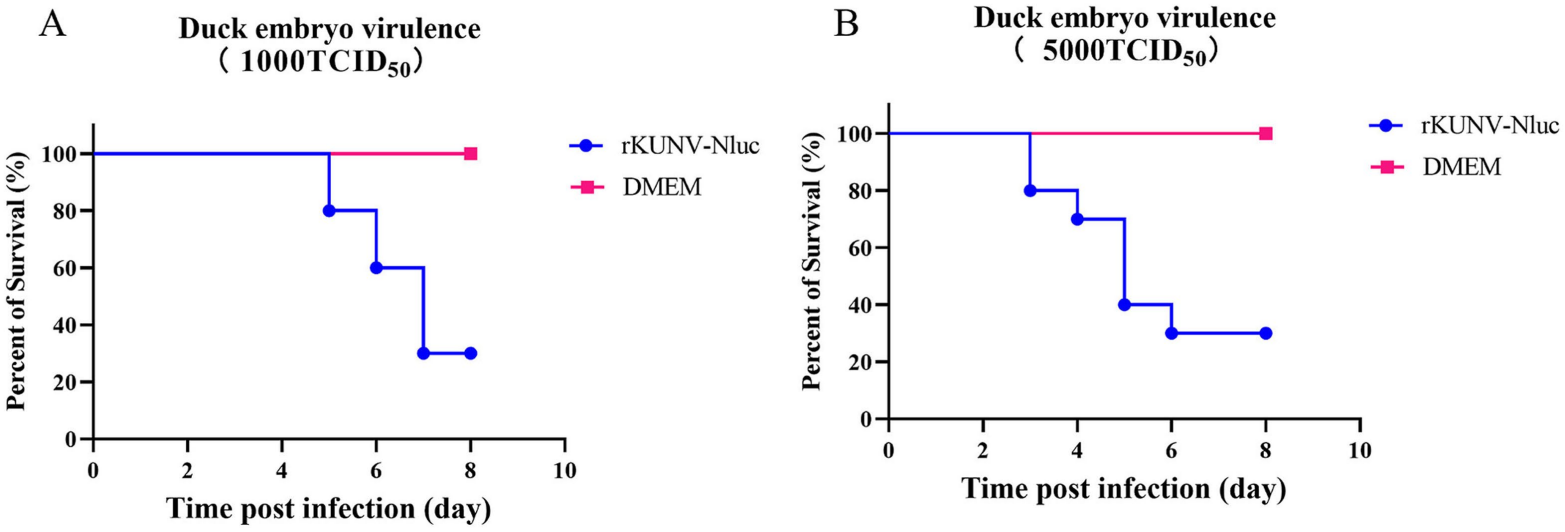
Virulence of the rKUNV-Nluc in duck embryos. **(A)** Virulence in duck embryos with 1,000 TCID_50_; **(B)** Virulence in duck embryos with 5,000 TCID_50_.

To assess virulence, 10-day-old embryonated duck eggs were inoculated via the allantoic route with 1 × 10^3 or 5 × 10^3 TCID₅₀ of rKUNV-Nluc. Embryos receiving 1 × 10^3 TCID₅₀ exhibited initial mortality on day 5 post-inoculation (dpi), with survivors remaining at 8 dpi (Figure 6A). Inoculation with 5 × 10^3 TCID₅₀ advanced the first mortality event to 3 dpi, though survivors persisted until the terminal observation point (Figure 6B). These findings demonstrate that rKUNV-Nluc elicits limited virulence in the duck embryo model, characterized by delayed onset mortality and incomplete lethality across both challenge doses.

## Discussion

Flavivirus replicon is a viral subgenome that has infectious defects but does not lose the ability to replicate, which is widely used in the research of flaviviruses (22–24). Flaviviruses have developed many replicons, but most were controlled by SP6 or T7 promoters and require *in vitro* transcription. However, DNA-based replicon exists in the form of plasmid, which could be directly transfected into cells without *in vitro* transcription (25), and is relatively stable and effectively absorbed by the host (26). Previous studies have shown that the first 20 amino acids of the C protein contain essential elements for genome replication and are therefore essential (27), and the C-terminal 24 residues of the E protein serve as the signal sequence of the NS1 protein and need to be retained (28). Therefore, in this study, a series of DNA-based reporter replicon were constructed by using pCC1-KUNV-rep as a template under the control of the CMV promoter, which retained the complete C protein and E35 sequence. HDVr and SV40 signal sequences were introduced downstream of the 3’UTR of the KUNV reporter replicon to generate authentic 3’ends and promote replication of the replicon (29, 30), and a GDD-AAA mutation was introduced into the NS5 gene to generate defective replicon with defective NS5 replication as a negative control. Functional characterization of KUNV reporter replicons *in vitro* showed that they could replicate in BHK-21, DEF, and LMH cells.

Packaging systems play an important role in the study of flavivirus life cycle, antiviral drug screening, and vaccine development (31–34).

In this study, co-transfection of helper plasmids expressing KUNV structural proteins with KUNV reporter replicon DNA generated a packaging system capable of packaging the KUNV replicon DNA, which generated SRIPs containing viral subgenomic replicon DNA that can be used to study processes such as viral assembly, release, and entry. We prepared two helper plasmids that expressed KUNV CprME and KUNV C_18_prME genes. When the CprME helper plasmid was co-transfected with the KUNV reporter replicon DNA, SRIPs packaging the KUNV replicon DNA were generated; however, the yield of infectious particles was rather low. This finding may be due to the significantly lower proportion of cells expressing both prME and C proteins or to the difference in replication efficiency of the two plasmids within the same cell. After co-transfection of C_18_prME helper plasmid and KUNV reporter DNA, the yield of infectious particles was greatly increased. When KUNV replicon does not provide prME protein, the KUNV replicon DNA is not packaged and does not produce any infectious particles, suggesting an important role for prME protein in particle assembly.

In addition, in this study, we also generated KUNV reporter virus by using Nluc luciferase as a reporter gene based on the infectious clone of KUNV. The Nluc luciferase possess a number of physical properties that make it an excellent marker for reporter virus construction. Our data demonstrate that the reporter virus that expressed the Nluc luciferase has a high replication fitness, that Nluc gene is highly expressed in infected mammalian and avian cells, that the gene produced a strong peak of luciferase activity, and that the inserted marker is fairly stable for at least five rounds of proliferation.

Operation of the reverse genetics system necessitates strict adherence to laboratory biosafety and containment protocols (35). Over the past several decades, reports have documented laboratory-acquired infections (LAIs) with WNV, including instances attributed to aerosol transmission and direct contact (36–38). Given that the reverse genetics system constructed in this study generates replication-competent live KUNV, all KUNV-related procedures must be performed under Biosafety Level 2 (BSL-2) containment practices, utilizing appropriate engineering controls and facility safeguards. Compared with natural viruses, pseudotyped viruses expressing C-prM/E or C_18_-prM/E contain deleted or modified sequences, typically replaced with reporter genes. As a result, they retain the ability to enter host cells but are incapable of producing infectious viral particles. Therefore, they do not pose an environmental risk and can be handled in laboratories below BSL-2 (39).

In summary, the reverse genetics platform for KUNV was successfully established in this study, including: KUNV reporter replicon, pseudovirus packaging system and KUNV reporter virus. The successful construction of the reporter replicon, packaging of SRIPs, and development of reporter virus provide a platform for further studies on flavivirus replication and the viral life cycle.

## Data Availability

The original contributions presented in the study are included in the article/supplementary material, further inquiries can be directed to the corresponding authors.

## References

[1] Scherret JH Poidinger M Mackenzie JS Broom AK Deubel V Lipkin WI. The relationships between West Nile and Kunjin viruses. Emerg Infect Dis. (2001) 7:697–705. doi: 10.3201/eid0704.017418, PMID: 11585535 PMC2631745

[2] Daffis S Lazear HM Liu WJ Audsley M Engle M Khromykh AA. The naturally attenuated Kunjin strain of West Nile virus shows enhanced sensitivity to the host type I interferon response. J Virol. (2011) 85:5664–8. doi: 10.1128/JVI.00232-11, PMID: 21411525 PMC3094947

[3] Hall RA Broom AK Smith DW Mackenzie JS. The ecology and epidemiology of Kunjin virus. Curr Top Microbiol Immunol. (2002) 267:253–69. doi: 10.1007/978-3-642-59403-8 PMID: 12082993

[4] Chambers TJ Hahn CS Galler R Rice CM. Flavivirus genome organization, expression, and replication. Ann Rev Microbiol. (1990) 44:649–88. doi: 10.1146/annurev.mi.44.100190.003245, PMID: 2174669

[5] Khromykh AA Westaway EG. Completion of Kunjin virus RNA sequence and recovery of an infectious RNA transcribed from stably cloned full-length cDNA. J Virol. (1994) 68:4580–8. doi: 10.1128/jvi.68.7.4580-4588.1994, PMID: 8207832 PMC236385

[6] Lindenbach BD. Virion assembly and release. Curr Top Microbiol Immunol. (2013) 369:199–218. doi: 10.1007/978-3-642-27340-7_8, PMID: 23463202 PMC3925669

[7] Zhang X Jia R Shen H Wang M Yin Z Cheng A. Structures and functions of the envelope glycoprotein in Flavivirus infections. Viruses. (2017) 9:338. doi: 10.3390/v9110338, PMID: 29137162 PMC5707545

[8] Villordo SM Gamarnik AV. Differential RNA sequence requirement for dengue virus replication in mosquito and mammalian cells. J Virol. (2013) 87:9365–72. doi: 10.1128/JVI.00567-13, PMID: 23760236 PMC3754043

[9] Gritsun TS Gould EA. Origin and evolution of 3'UTR of flaviviruses: long direct repeats as a basis for the formation of secondary structures and their significance for virus transmission. Adv Virus Res. (2007) 69:203–48. doi: 10.1016/S0065-3527(06)69005-2 PMID: 17222695

[10] Hall RA Scherret JH Mackenzie JS. Kunjin virus: an Australian variant of West Nile? Ann N Y Acad Sci. (2001) 951:153–60. doi: 10.1111/j.1749-6632.2001.tb02693.x 11797773

[11] Prow NA. The changing epidemiology of Kunjin virus in Australia. Int J Environ Res Public Health. (2013) 10:6255–72. doi: 10.3390/ijerph10126255, PMID: 24287851 PMC3881112

[12] Hall RA Khromykh AA Mackenzie JM Scherret JH Khromykh TI Mackenzie JS. Loss of dimerisation of the nonstructural protein NS1 of Kunjin virus delays viral replication and reduces virulence in mice, but still allows secretion of NS1. Virology. (1999) 264:66–75. doi: 10.1006/viro.1999.9956, PMID: 10544130

[13] Mackenzie JS Lindsay MD Coelen RJ Broom AK Hall RA Smith DW. Arboviruses causing human disease in the Australasian zoogeographic region. Arch Virol. (1994) 136:447–67. doi: 10.1007/BF01321074, PMID: 8031248

[14] Russell RC Dwyer DE. Arboviruses associated with human disease in Australia. Microbes Infect. (2000) 2:1693–704. doi: 10.1016/S1286-4579(00)01324-1, PMID: 11137043

[15] Naim H Wild J Boughton CR Hawkes RA. Arbovirus surveillance in the Murray-Riverina region (1982) 82:5–6.

[16] Kay BH Boreham PF Fanning ID. Host-feeding patterns of Culex annulirostris and other mosquitoes (Diptera: Culicidae) at Charleville, southwestern Queensland, Australia. J Med Entomol. (1985) 22:529–35. doi: 10.1093/jmedent/22.5.529, PMID: 2864452

[17] Marshall ID. Murray valley and Kunjin encephalitis. Arboviruses. (2019) 4:151–90. doi: 10.1201/9780429280276-9

[18] Rossi SL Ross TM Evans JD. West Nile virus. Clin Lab Med. (2010) 30:47–65. doi: 10.1016/j.cll.2009.10.006, PMID: 20513541 PMC2905782

[19] Chen S He Y Zhang R Liu P Yang C Wu Z. Establishment of a reverse genetics system for duck Tembusu virus to study virulence and screen antiviral genes. Antivir Res. (2018) 157:120–7. doi: 10.1016/j.antiviral.2018.06.016, PMID: 30057296

[20] Wu Z Hu T Zhou Z He Y Wang T Wang M. First establishment of a duck model for in vivo and in vitro studies of west nile virus (Kunjin subtype). Poult Sci. (2025) 104:105214. doi: 10.1016/j.psj.2025.105214, PMID: 40315579 PMC12098138

[21] He Y Liu P Wang T Wu Y Lin X Wang M. Genetically stable reporter virus, subgenomic replicon and packaging system of duck Tembusu virus based on a reverse genetics system. Virology. (2019) 533:86–92. doi: 10.1016/j.virol.2019.05.003, PMID: 31136895

[22] Shi PY Tilgner M Lo MK. Construction and characterization of subgenomic replicons of New York strain of West Nile virus. Virology. (2002) 296:219–33. doi: 10.1006/viro.2002.1453, PMID: 12069521

[23] Alcaraz-Estrada SL Del Angel R Padmanabhan R. Construction of self-replicating subgenomic dengue virus 4 (DENV4) replicon. Methods Mol Biol. (2014) 1138:131–50. doi: 10.1007/978-1-4939-0348-1_9, PMID: 24696335

[24] Manokaran G McPherson KG Simmons CP. Attenuation of a dengue virus replicon by codon deoptimization of nonstructural genes. Vaccine. (2019) 37:2857–63. doi: 10.1016/j.vaccine.2019.03.062, PMID: 31000413

[25] Varnavski AN Young PR Khromykh AA. Stable high-level expression of heterologous genes in vitro and in vivo by noncytopathic DNA-based Kunjin virus replicon vectors. J Virol. (2000) 74:4394–403. doi: 10.1128/JVI.74.9.4394-4403.2000, PMID: 10756054 PMC111956

[26] Huang Q Yao Q Fan H Xiao S Si Y Chen H. Development of a vaccine vector based on a subgenomic replicon of porcine reproductive and respiratory syndrome virus. J Virol Methods. (2009) 160:22–8. doi: 10.1016/j.jviromet.2009.04.004, PMID: 19464739

[27] Khromykh AA Westaway EG. Subgenomic replicons of the flavivirus Kunjin: construction and applications. J Virol. (1997) 71:1497–505. doi: 10.1128/jvi.71.2.1497-1505.1997, PMID: 8995675 PMC191206

[28] Jones CT Patkar CG Kuhn RJ. Construction and applications of yellow fever virus replicons. Virology. (2005) 331:247–59. doi: 10.1016/j.virol.2004.10.034, PMID: 15629769

[29] Pierson TC Sánchez MD Puffer BA Ahmed AA Geiss BJ Valentine LE. A rapid and quantitative assay for measuring antibody-mediated neutralization of West Nile virus infection. Virology. (2006) 346:53–65. doi: 10.1016/j.virol.2005.10.030, PMID: 16325883

[30] Boyer JC Haenni AL. Infectious transcripts and cDNA clones of RNA viruses. Virology. (1994) 198:415–26. doi: 10.1006/viro.1994.1053, PMID: 8291226

[31] Lu C-Y Hour MJ Wang CY Huang SH Mu WX Chang YC. Single-round infectious particle antiviral screening assays for the Japanese encephalitis virus (2017) 9:76. doi: 10.3390/v9040076, PMID: 28394283 PMC5408682

[32] Lin CS Li WJ Liao CY Kan JY Kung SH Huang SH. A reverse mutation E143K within the PrM protein of Zika virus Asian lineage Natal RGN strain increases infectivity and Cytopathicity. Viruses. (2022) 14:572. doi: 10.3390/v14071572, PMID: 35891552 PMC9317194

[33] Huang YT Liao JT Yen LC Chang YK Lin YL Liao CL. Japanese encephalitis virus replicon-based vaccine expressing enterovirus-71 epitope confers dual protection from lethal challenges. J Biomed Sci. (2015) 22:74. doi: 10.1186/s12929-015-0181-8, PMID: 26362772 PMC4566489

[34] Kobayashi D Inoue Y Suzuki R Matsuda M Shimoda H Faizah AN. Identification and epidemiological study of an uncultured flavivirus from ticks using viral metagenomics and pseudoinfectious viral particles. Proc Natl Acad Sci USA. (2024) 121:e2319400121. doi: 10.1073/pnas.2319400121, PMID: 38687787 PMC11087778

[35] Berankova M Leoni S Holoubek J Haviernik J Salat J Grandgirard D. Three-dimensional mapping of tick-borne encephalitis virus distribution in the mouse brain using a newly engineered TurboGFP reporter virus. Emerg Microbes Infect. (2025) 31:2542246. doi: 10.1080/22221751.2025.2542246 PMC12442459 40743437

[36] Laboratory safety for arboviruses and certain other viruses of vertebrates. The subcommittee on arbovirus laboratory safety of the American committee on arthropod-borne viruses. Am J Trop Med Hyg. (1980) 29:1359–81. doi: 10.4269/ajtmh.1980.29.1359, PMID: 6778230

[37] Nile W. Laboratory-acquired West Nile virus infections-United States. MMWR Morb Mortal Wkly Rep. (2002) 51:1133–5. 12537288

[38] Venter M Burt FJ Blumberg L Fickl H Paweska J Swanepoel R. Cytokine induction after laboratory-acquired West Nile virus infection. N Engl J Med. (2009) 360:1260–2. doi: 10.1056/NEJMc0808647, PMID: 19297584

[39] Zhang L Wang X Ming A Tan W. Pseudotyped virus for Flaviviridae. Adv Exp Med Biol. (2023) 1407:313–27. doi: 10.1007/978-981-99-0113-5_17, PMID: 36920705

